# Area-level deprivation, neighbourhood factors and associations with mental health

**DOI:** 10.1371/journal.pone.0281146

**Published:** 2023-01-30

**Authors:** Gretta Mohan, Peter Barlow

**Affiliations:** 1 Economic and Social Research Institute, Dublin, Ireland; 2 Department of Economics, Trinity College, Dublin, Ireland; 3 University of Edinburgh, Edinburgh, Scotland, United Kingdom; University of Georgia, UNITED STATES

## Abstract

The COVID-19 pandemic saw residential neighbourhoods become more of a focal point in people’s lives, where people were greater confined to living, working, and undertaking leisure in their locality. This study investigates whether area-level deprivation and neighbourhood conditions influence mental health, accounting for demographic, socio-economic and health circumstances of individuals. Using nationally representative data from Ireland, regression modelling revealed that area-level deprivation did not in itself have a discernible impact on mental health status (as measured using the Mental Health Inventory-5 instrument and the Energy and Vitality Index), or likelihood of having suffered depression in the previous 12 months. However, positive perceptions of area safety, service provision, and area cleanliness were associated with better mental health, as was involvement in social groups. Broad ranging policies investing in neighbourhoods, could have benefits for mental health, which may be especially important for deprived communities.

## Introduction

The impact of area-level deprivation on public health, health status and health outcomes has attracted significant interest from healthcare policymakers and researchers. Following a landmark US Surgeon General report in 1999, the importance of a nation’s mental health was underscored for policy, where mental health was defined as “a state of successful performance of mental function, resulting in productive activities, fulfilling relationships with other people, and the ability to adapt to change and to cope with adversity” [1]. The World Health Organization (WHO) has championed the promotion and protection of mental health, asserting in its *Comprehensive Mental Health Action Plan 2013–2030* that “mental health is an integral part of health and wellbeing” [2]. Furthermore, the WHO has recognised that neighbourhoods can be key places for the promotion of mental health and wellbeing [3], and neighbourhood deprivation has been identified as a social determinant of mental health [4].

The study setting of this investigation is Ireland, where a series of national Irish governments have recognised the importance of promoting mental health and providing appropriate mental healthcare to those in deprived areas. The organisation responsible for the delivery of healthcare services, the Department of Health, recently published an updated mental health strategy, *Sharing the Vision* [5], which builds on an original strategy published in 2006 by the Irish national administrative health body, the Health Service Executive [6]. In acknowledging a potential influence of locality on mental health, the new strategy aims to deliver “a range of integrated activities to promote positive mental health in the community”, and outlines an objective that “services become community based” [5]. In addition, the national suicide prevention strategy suggests that there is a need to increase engagement with “disadvantaged families and communities” [7], where a disparity in mental health outcomes between deprived and affluent areas has been evidenced by an increased prevalence of suicide in deprived areas in Ireland [8]. In the context of this research, the terms ‘disadvantaged’ and ‘deprived’ areas relate to neighbourhoods in which residents typically have lower incomes, but these terms also encompass considerations such as fewer resources or opportunities in areas e.g., higher unemployment rates, lower skills attainment. A study of neighbourhoods and mental health in the setting of Ireland may also be interesting in the international context as Ireland has been found to have less residential segregation of neighbourhoods in terms of migration, ethnicity and other social indicators compared to other Western European countries and the United States [9].

This paper is motivated to assess the effect of living in a deprived community on the mental health of resident adults, to contribute to the international debate on these associations and to guide policymaking and healthcare planning in Ireland and internationally. The mental health of individuals living in an area is of policy relevance since it has important implications for both individuals and wider society. A persons’ mental health state has been demonstrated to affect productivity [10], and overall quality of life and physical health [11]. In the US, the cost per individual with major depression disorder has been estimated as $6524 for 2018, with the national economic burden of adults with major depression disorder estimated at $326.2 billion (encompassing direct medical and pharmaceutical-related costs, suicide-related costs, and workplace costs) [12]. For the study setting of Ireland, the annual economic cost of mental health problems has been estimated as €11 billion [13]. As such, the wider costs to society associated with mental health challenges, provide a justification for studying factors such as environmental influences which could influence mental health and, potentially, be a channel through which government policy and intervention can be deployed to improve outcomes.

Moreover, recent experiences of government mandated lockdowns during the COVID-19 pandemic, which restricted movements of residents to within limited boundaries of their localities, have been linked to deleterious effects on mental health. A study from Japan found that deprived and more urban areas had greater levels of psychological distress and had poorer mental health during COVID-19 [14]. On the other hand, a US study found that a lack of negative neighbourhood conditions (e.g., crime, violence and traffic) were associated with a lower risk of mental health difficulties during the pandemic including depression, anxiety and loneliness [15]. In a systematic review of the effects of the natural environment on mental health, which included studies that covered the period of the COVID-19 pandemic, Lanza-León et al. [16], concluded that exposure to, use and proximity to green spaces had differential effects on mental health across the groups studied. Beneficial impacts were found for the mental health of the elderly, students and patients with underlying pathologies, while negative effects on the mental health of women and young adults were discovered.

In this investigation, we study whether area-level deprivation, as measured by an objective index which accounts for multiple-forms of deprivation in a defined geographic area, has an influence on three different metrics of mental health: positive mental health, negative mental health, and depression. The overall aim of the work is to contribute to an active scholarly debate concerned with the influence of area-level deprivation, and aspects of neighbourhood, on mental health. To the best of the author’s knowledge, the relationship between mental health and neighbourhood circumstances has not been explored in the context of Ireland, and thus may inform domestic Irish policymaking.

The remainder of the paper is structured as follows. The subsequent section discusses the literature examining associations between neighbourhood and mental health. This is followed by a description of the data and methods used for this analysis. The results are presented. The implications of the findings for research and policymaking communities are considered in a discussion section, drawing conclusions.

## Literature review

The role of place in one’s health is a matter which has attracted increased consideration in health and epidemiological literature [17–19]. A relatively early study [20], indicated that poor housing quality, neighbourhood quality and residing in the ground floor of an apartment building may have negative implications for an individual’s mental health. However, the empirical evidence that examines the effects of neighbourhoods on health which has since emerged paints a mixed picture as to the extent of an effect, calling for further studies on place effects [19]. A systematic review of 99 studies concluded that there was a lack of evidence of the strength or presence of effects of the built environment in which an individual resides on mental health [21].

On the other hand, a substantial body of papers have found evidence for an environmental effect from area-level deprivation on mental health. A systematic review of 14 longitudinal studies found that greater deprivation levels in neighbourhoods were associated with increased depressive symptoms [22]. The authors propose that disadvantaged communities may also be more vulnerable to stress due to a lack of communal resources despite having strong social supports.

Neighbourhood deprivation was linked with increased levels of depression based on an analysis of adult twins and found that it modified the initial genetic risk of acquiring depression [23]. How neighbourhoods interact with people’s environment has also been found to affect a person’s likelihood of presenting with depressive symptoms [24]. For example, individuals in areas with a higher density of auto commuters (presumably those who commute by car), were more likely to experience depressive symptoms. A review of 28 papers which examined the impact of neighbourhood social deprivation and psychotic disorders found that there was consistent evidence of greater psychotic problems in more socially deprived areas [25]. Negative mental health was also found to be correlated with increased area-level problems and worse social cohesion in the US [26]. Moreover, a systematic review of the relationship between socio-economic deprivation and the occurrence of suicide, found that for 25 out of 27 studies a greater level of suicidal behaviour was reported for more deprived areas [27].

Empirical support for a link between negative mental health, measured by the Mental Health Inventory (MHI) 5 index, and area-level deprivation, measured by the number of benefits recipients at the ward level in an area of Wales, UK, has been documented [28]. A prior investigation into mental health inequalities amongst communities in Wales found that regional socio-economic disparity explained a proportion of the difference in mental health whereby more deprived areas had worse mental health [29].

In a study of a US nationwide dataset, neighbourhood disadvantage was revealed to have an overall negative impact on mental health, and this was related to mental health in three ways [30]. Firstly, neighbourhood deprivation was directly linked to depression. Second, area-level deprivation increased depression through a greater level of stressors such as drug use, alcohol use and crime in the area. Third, the effect of neighbourhood deprivation was mitigated by enhanced social supports such as whether an individual had someone ‘to lean on, to talk to or to help with a task’ in disadvantaged communities. Evidence concerning area-level deprivation and the utilisation of healthcare in Ireland has also found that there is greater utilisation of General Practitioner (GP) services in disadvantaged areas [31].

On the other hand, there is also a corpus of evidence for which no link between area-level deprivation and mental health has been established. A study found that there was little evidence that area-level socio-economic deprivation itself affected common mental disorders, but household-level social factors were significant [32]. The social factors detailed included overcrowding, household type, housing tenure and structural housing problems. Research using survey data on the general population of the city of Amsterdam, found that while there was no impact of area-level deprivation on one’s propensity to have major mental disorders, the collection of individuals of lower socioeconomic status in deprived areas resulted in more cases of mental disorder in these areas [33].

A study employing the British Household Panel Survey, found that individual characteristics such as whether an individual was white or of other ethnicity, determined an individual’s propensity for mental disorder rather than the characteristics of place [34]. In a subsequent investigation, analysing British renters longitudinally over a ten year period, it was revealed that residing in neighbourhoods consisting of more individuals in poverty had no effect on a person’s personal income or their individual mental health [35]. Perceptions of mental health, and economic, social and cultural factors affecting this has also emerged as important [36]. In a qualitative study, those who resided in a deprived inner-city area of London were more positive about their neighbourhood than outsiders, and they did not perceive that living in their area was bad for their health [37].

Within the literature exploring the association between area-level deprivation and mental health, there is typically a recognition of, and focus on, a greater presence of environmental stressors in deprived areas, including an increased fear of crime, litter and poverty, and it is these which are associated with negative impacts on an individual’s mental and physical health [38].

A review of the literature assessing the causal pathways from area-level deprivation to increased depression, found that social processes were of greater importance in affecting increased depressive symptoms when compared to the socio-economic or racial composition of neighbourhoods [39]. It has also been suggested that area-level deprivation created more stressors for individuals who resided in those areas [40]. Increased stressors, for example, greater prevalence of crime in an area or poorer quality of housing, have been shown to have negative implications for a mental health [41].

On the other hand, social supports within a community have been found to mediate the relationship between mental health and area-level deprivation. Individuals with high levels of social capital in areas of high deprivation have been found to have a reduced likelihood of experiencing common mental disorders [42]. A study found that residing in a neighbourhood of higher deprivation in the Netherlands was associated with poorer mental, irrespective of socio-demographic characteristics and the presence of chronic illness [43]. However, the association between neighbourhood deprivation and mental health-related quality of life was only observed among persons with few personal contacts or low social need fulfilment, suggesting that social relations buffered the effect of neighbourhood deprivation. Analysing 32 neighbourhoods in the US, it was also discovered that social networks mediated the relationship between area-level deprivation and depressive symptoms [44].

In summary, the literature which examines the relationship between area-level deprivation and mental health presents a myriad of findings though a common theme finds deprived areas to be more stressful and living in these may have implications for mental health. We also note a large variety of measures of mental health and neighbourhood deprivation used across studies to assess associations. The research presented in this paper examines mental health from multiple different angles, encompassing positive and negative mental health measures and depression status, to provide a holistic, rounded study of effects on mental health. Within this same study, multiple area-level factors which are thought to influence mental health, identified across the range of extant literature, including area-level safety, cleanliness and service provision are examined. As such, the work adds a more comprehensive investigation of these relationships in the study setting of Ireland.

## Data and methods

### Data

*Healthy Ireland* is a nationally representative, cross-sectional survey which captured data on demographic, socioeconomic, and health information of 7,498 respondents in 2016 and informs this study. The 2016 survey is used for this analysis as only the 2016 questionnaire inquired about respondent’s local neighbourhood and social connectedness, in addition to core questions on a person’s health and wellbeing, and health behaviours. The neighbourhood questions were included as a one-off theme of interest, while surveys since then have enquired as to other one-off health-related topics e.g., sun protection, sleep, menstrual health etc. Data collection for *Healthy Ireland* 2016 was carried out via in-person face to face interviews, conducted by a market research company, Ipsos MRBI, on behalf of Ireland’s Department of Health [45]. The response rate for 2016 was 59.9%.

As data from the *Healthy Ireland* survey is accessed as a secondary data source, ethical approval for this study was not necessary. Ethical approval for the collection of *Healthy Ireland* data was provided by the Research Ethics Committee at the Royal College of Physicians of Ireland.

#### Dependent variables

*Healthy Ireland* asks a series of questions relating to mental health which provide three suitable metrics of mental health status for this investigation. The first, poor mental health, is captured by the Mental Health Inventory index (MHI-5); a second indicator concerns positive mental health, derived from the Energy and Vitality Index (EVI) instrument; and the third indicator relates to a question concerning whether an individual had suffered with depression in the previous 12 months. As such, these three different indicators of mental health attempt to provide as rounded as possible view of the state of mental health. Further details on the outcomes are provided below.

*Poor mental health*: *MHI-5*. The MHI-5 is a standard measure of negative mental health which originates from the RAND SF-36 questionnaire [46]; an aggregated score from five questions concerning an individual’s mental state in the past seven days is derived, listed in Table 1. The respondent may choose a response to each of the five questions from one of six possible options, which refer to the amount of time that the respondent felt the particular mental state in question over the previous week as: ‘All of the time’, ‘Most of the time’, ‘A good bit of the time’, ‘Some of the time’, ‘A little of the time’ or ‘None of the time’. Based on the responses, a 5-point score is created for each of the variables. In these questions, a score of 5 is associated with having poorer mental health more of the time. These are combined for each of the five questions to provide a score of negative mental health between 0 and 100. A higher score indicates poorer mental health.

**Table 1:** MHI-5 and EVI items.

In the past 7 days,	
**MHI-5 Index item**	1. how often have you been a very nervous person?
	2. how often have you felt so down in the dumps that nothing could cheer you up?
	3. how often have you felt calm and peaceful?
	4. how often have you felt downhearted and low?
	5. how often have you been a happy person?
**EVI Index item**	1. how often did you feel full of life?
	2. how often have you felt calm and peaceful?
	3. how often did you feel worn out?
	4. how often did you feel tired?

Response options:

All of the time; Most of the time; A good bit of the time; Some of the time; A little of the time; or None of the time.

*Positive mental health*: *EVI*. The EVI indicator provides a score derived from four questions which inquire about a person’s level of energy and mental attitude to life, the items for which are listed in Table 1. Similar to the MHI5, the EVI asks how the individual has felt or been in the past seven days, with six possible responses ranging from ‘All of the time’ to ‘None of the time’. The response to each of the four questions are then combined to a scale of 0–100, indicating their level of positive mental health. A higher score indicates better positive mental health. The EVI also originates from the RAND SF-36 questionnaire [46], and, in a study of mental health indicators for Europe, the EVI was recommended as an effective proxy for positive mental health [47]. The EVI has been employed in the Irish context to measure population mental health and social wellbeing [48].

*Depression*. Survey respondents were asked to report whether they had suffered with depression within the previous 12 months. The indicator is a self-report, with a dummy value of 1 indicating that the respondent had suffered with depression, 0 otherwise.

*Exposure variables*. This paper examines the impact of neighbourhood on an individual’s mental health. A number of variables were available from the 2016 *Healthy Ireland* survey which provide a picture of the nature of a respondent’s area of residence. The primary exposure of interest is an objective indicator of the level of deprivation in the neighbourhood, measured by the Haas Pratschke (HP) index. Secondary exposures of interest include reported levels of neighbourhood safety, cleanliness and service provision. We also assess whether social connectedness, as indicated by membership of a social club, is associated with mental health outcomes.

*Area-level deprivation*: *HP index*. The HP deprivation index associated with the neighbourhood of residence of the *Healthy Ireland* respondent provides an objective measure of area-level deprivation, where the index has been specifically tailored for the context of Ireland. The construction of the HP index is similar to other deprivation indexes such as the Index of Multiple Deprivation used in the UK [49, 50]. The HP Index uses confirmatory factor analysis to develop a measure of deprivation for small areas based on their social class composition, labour market conditions and demographic conditions. Built on these three factors [51], informed by small area statistics from Ireland’s census (2011), the HP index constructs a score for the relative deprivation of each small area in Ireland (the smallest area for which the Central Statistics Office provides data, comprising of between 80 and 120 dwellings). A lower HP score implies greater deprivation, and for the purposes of this research, the HP scores of the neighbourhood of respondents are divided into quintiles of deprivation levels. Quintile one is the most deprived quintile; while quintile five is the least deprived, most affluent quintile. Further details on the development of the HP index are provided in the S1 File.

*Other area-level variables*. The 2016 survey of *Healthy Ireland* enquired about the existence of a number of problems in the neighbourhood of respondents, which could be grouped into three categories: area-level safety, cleanliness, and service provision, summarised in Table 2. We note that the definition of neighbourhood is left as subjective to the respondent–the concept of neighbourhood is self-interpreted by the respondent and not defined by an objective measure e.g., postcode or street level. Respondents were asked the degree to which each item was a problem in their neighbourhood, to which they could respond ‘A big problem’, ‘A bit of a problem’ and ‘Not a problem’.

**Table 2:** Grouping of variables capturing perceptions of area-level problems.

Area safety	Area cleanliness	Area service provision
Public drunkenness Racism House break-ins	Vandalism Rubbish/litter Graffiti	Public transport Food shops Open spaces

### Method

#### Ordinary Least Squares (OLS) Regression—MHI-5 and EVI

To estimate the effect of neighbourhood deprivation and area-level factors on the continuous dependent variables of negative mental health (as measured by the MHI-5 index) and positive mental health (indicated by EVI), a conventional OLS regression is employed. This method assumes a linear relationship between area-level deprivation, neighbourhood factors and the individual’s mental health; the relationships for estimation may be represented by Eqs 1, 2 and 3:

$$MHS_{i}=\beta_{Dep}X_{i,Dep}+\beta_{d}X_{i,d}$$ (1)

$$MHS_{i}=\beta_{Dep}X_{i,Dep}+\beta_{d}X_{i,d}+\beta_{se}X_{i,se}$$ (2)

$$MHS_{i}=\beta_{Dep}X_{i,Dep}+\beta_{d}X_{i,d}+\beta_{se}X_{i,se}+\beta_{A}X_{i,A}.$$ (3)

In Eqs 1, 2 and 3, the dependent variable, the mental health score is represented by *MHS*_*i*_. The estimated influence of area-level deprivation on mental health is represented by *β*_*Dep*_, where *X*_*i*,*Dep*_ is the quintile of deprivation of the residential area where the individual resides. This effect is examined with reference to the base category of residing in the least deprived quintile (quintile 5). The most basic model is represented by Eq (1), where the influence of area-level deprivation on mental health also includes an adjustment for demographic controls, *X*_*i*,*d*_, specifically, gender and age. A further adjusted model, Eq (2) adds additional controls to the base model (1), including socio-economic and health variables, *X*_*i*,*se*_, specifically, marital status, educational attainment, employment status, urbanity, whether the survey respondent has had a long term illness, self-reported health, public health insurance (i.e.) medical card status, whether they were a smoker, whether they were an immigrant, and whether the respondent reports membership of a social group/club. A final model, (3), adjusts model (2) to account for area-level problems, *X*_*i*,*A*_, including problems with area-level cleanliness, safety, and service provision. We note that due to data privacy and anonymity reasons the dataset does not contain information on the location of respondents to allow for clustering at small geographical areas, nor multi-level analysis, and thus robust standard errors are employed in models.

#### Logistic regression—Depression

To estimate the impact of area-level deprivation and other variables on the binary variable of whether a person reported experiencing depression in the previous 12 months, a logistic regression model is employed. This facilitates the calculation of the marginal effect of living in a deprived area or an area with various neighbourhood problems on an individual’s likelihood of having experienced depression. The models corresponding to Eqs 1, 2 and 3 for the mental health score measures above are applied to the binary outcome of depression, illustrated in Eqs 4, 5 and 6:

$$P\left(Y_{Depression}=1\right)=\frac{\text{exp}(\beta_{Dep}X_{Dep}+\beta_{D}X_{d})}{1+\;\text{exp}(\beta_{Dep}X_{Dep}+\beta_{D}X_{d})}$$ (4)

$$P\left(Y_{Depression}=1\right)=\;\frac{\text{exp}(\beta_{Dep}X_{Dep}+\beta_{D}X_{d}+\beta_{se}X_{se)}}{1+\;\text{exp}(\beta_{Dep}X_{Dep}+\beta_{D}X_{d}+\beta_{se}X_{se})}$$ (5)

$$P\left(Y_{Depression}=1\right)=\frac{\text{exp}(\beta_{Dep}X_{Dep}+\beta_{D}X_{d}+\;\beta_{se}X_{se}\;+\;\beta_{A}X_{A})}{1+\;\text{exp}(\beta_{Dep}X_{Dep}+\beta_{D}X_{d}+\beta_{se}X_{se}\;+\beta_{A}X_{A})}$$ (6)

The outcome is symbolised as *Y*_*Depression*_, where the probability of whether an individual reported suffering with depression is represented as *P*(*Y*_*Depression*_ = 1). The exponential of the natural logarithm is represented in the equation by *exp*. Of the 7,498 individuals surveyed in 2016, 7,403 provided responses to all the questions used in the analysis for this study and the analysis is performed on the complete case sample.

#### Principal components analysis

To ascertain the importance of different aspects of an individual’s neighbourhood on their mental health, several variables relating to people’s perceptions of their area are of interest. However, where all of these variables are individually included in models simultaneously, this could lead to issues with the multi-collinearity [52]. Therefore, to reduce the number of variables included in the models, and the potential for multicollinearity to affect estimated effects, principal component analysis was applied to the perceived area-level variables grouped in Table 3 [53]. The first principal component of each group was taken to estimate variables that indicate ‘area-level safety’, ‘area-level cleanliness’ and ‘area-level service provision’. This facilitated an analysis of the impact of the various latent variables of perceptions of area-level safety, perceptions of area-cleanliness, and perceptions of area-service provision on an individual’s mental health.

**Table 3:** Scoring coefficients from principal components analysis.

Area-level safety	Scoring coefficient
Public drunkenness	0.619
Racism	0.481
House break-ins	0.621
*Eigenvalue*	*1*.*625*
**Area-level cleanliness**	
Vandalism	0.588
Rubbish	0.555
Graffiti	0.589
*Eigenvalue*	*2*.*021*
**Area-level service provision**	
Problem with public transport	0.642
Problem with food shops	0.653
Problem with open space	0.401
*Eigenvalue*	*1*.*589*

Table 3 documents the scoring coefficients of the variables derived from the principal component analysis and the variables on which it is based. These coefficients indicate the direction and magnitude of the correlation between the principal component and the composite variables. For example, in Table 3, the variable ‘house break-ins’ is strongly positively correlated with ‘area-level safety’ (0.621). Eigenvalues represent the total amount of variance that can be explained by a given principal component, where an eigenvalue greater than 1 indicates that a principal component accounts for more variance than that accounted for by one of the original variables.

*Potential differential group influences*. We also analyse whether there is a differential effect of the neighbourhood level variables of interest on an individual’s mental health by the subgroups of gender and urban location. To examine this, interaction variables of gender and urban location with the deprivation quintiles are examined within the models of the main analysis.

*Alternative model specification as checks of the results*. Several robustness analyses were conducted, including a logistic analysis of whether survey respondents had a MHI-5 or EVI score which exceeded a threshold of 80 (out of 100), and an alternative Probit regression approach was used to assess depression diagnosis outcome.

## Results

**Fig 1:**
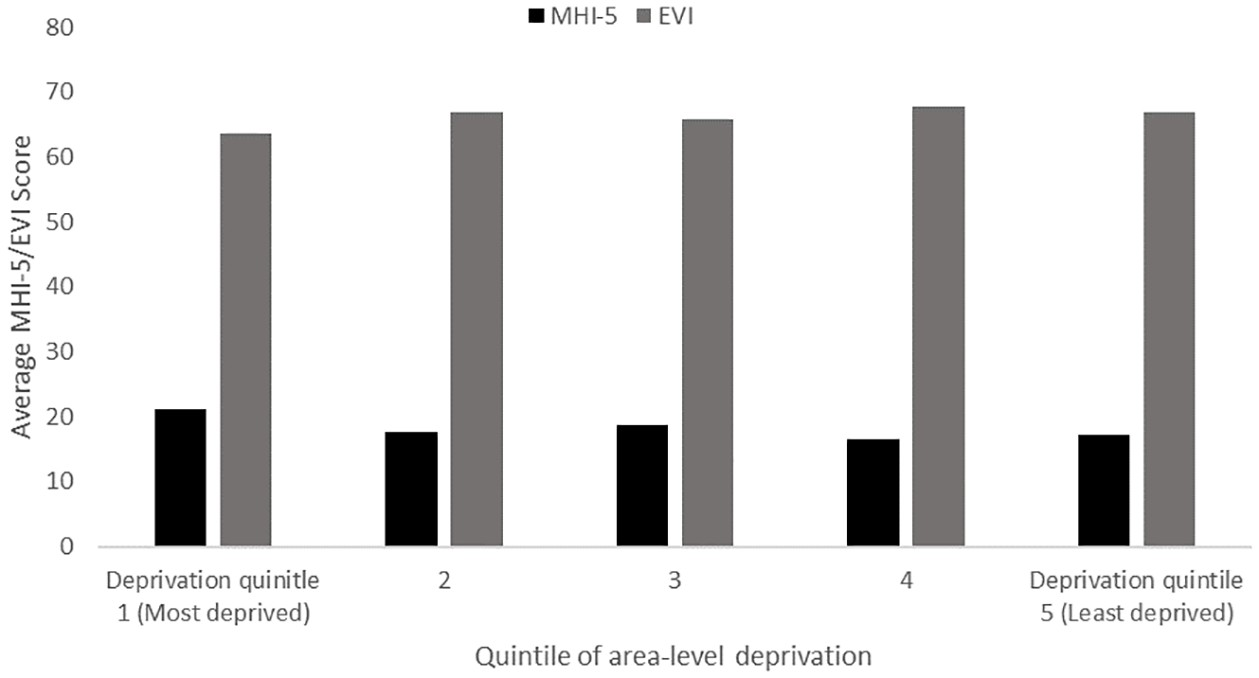
Average poor mental health score (MHI-5) and positive mental health score (EVI), by quintile of area-level deprivation.

**Fig 2:**
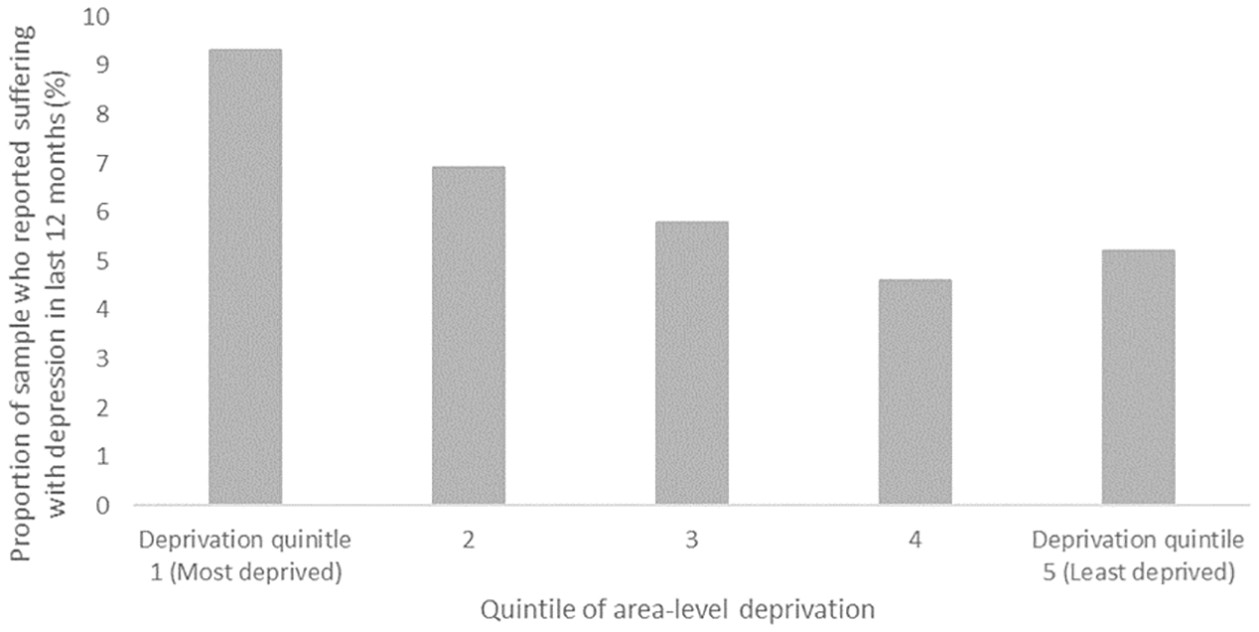
Percentage of respondents with depression, by quintile of area-level deprivation.

For the sample of analysis, Fig 1 demonstrates that mean scores of the MHI-5 and EVI4 indicators of mental health do not differ substantially across the various quintiles of area-level deprivation. On the other hand, Fig 2 displays a slight gradient in the proportion of the sample reported having suffered with depression by quintile of deprivation; where in the most deprived quintile the proportion of respondents with depression was 9.3%, decreasing across deprivation quintiles to 4.6% for the second least deprived quintile, increasing to 5.2% for the least deprived quintile.

The summary statistics which describe the characteristics of the sample are presented in Table 4, where the average MHI-5 score for the whole sample was 18.3, the average EVI was 66.2, and 6.5% of the sample reported having suffered with depression. In terms of the makeup of the sample, females accounted for 55.8%, and 41.3% of the population completed tertiary education. Less than half of the sample reported membership of a social group or club (44.0%).

**Table 4:** Summary statistics, sample for analysis.

**Observations**					7,403
**Variable (Continuous)**		**Mean**	**Standard Deviation**	**Minimum**	**Maximum**
*Poor mental health*: *MHI-5*		18.3	15.6	0	100
*Positive mental health*: *EVI4*		66.2	19.6	0	100
**Variable (Categorical)**	**Category**				**Percentage of sample (%)**
*Suffered from depression in previous 12 months*	Yes				6.5
	No				93.5
*Area-level deprivation quintile*	Quintile 1 (Most deprived)				21.1
	Quintile 2				22.3
	Quintile 3				19.6
	Quintile 4				20.1
	Quintile 5 (Least deprived)				16.9
*Age category*	15–24				8.2
	25–44				32.9
	45–64				32.8
	65 or greater				26.2
*Gender*	Male				44.2
	Female				55.8
*Marital status*	Married				52.5
	Unmarried				47.5
*Education*	Primary				11.4
	Secondary				47.3
	Tertiary				41.3
*Urban*	Urban				61.4
	Rural				38.6
*Public health insurance (medical card status)*	No medical card				53.8
	GP visit card				5.9
	Medical card				40.3
*Private health insurance status*	Insured				47.7
	Uninsured				52.3
*Employment status*	Employed				47.1
	Unemployed				6.3
	Student				22.1
	Retired				24.5
*Immigrant*	Yes				16.1
	No				83.9
*Self-rated health*	Good or very good				81.9
	Fair, poor or very poor				18.1
*Any long-term illness*	Yes				31.6
	No				68.4
*Membership of social groups/clubs*	Yes				44.0
	No				56.0
*Problem with*: *Public drunkenness in area*	No problem				89.3
	A bit of a problem				8.6
	A big problem				2.2
*Racism in area*	No problem				95.0
	A bit of a problem				3.7
	A big problem				1.3
*House break-ins in area*	No problem				65.3
	A bit of a problem				28.0
	A big problem				6.7
*Vandalism in area*	No problem				87.0
	A bit of a problem				10.6
	A big problem				2.4
*Rubbish/litter in area*	No problem				73.3
	A bit of a problem				20.5
	A big problem				6.2
*Graffiti in area*	No problem				89.2
	A bit of a problem				8.9
	A big problem				1.9
*Poor public transport in area*	No problem				18.6
	A bit of a problem				15.6
	A big problem				65.9
*Lack of food shops in area*	No problem				83.6
	A bit of a problem				11.3
	A big problem				5.1
*Provision of open space in area*	No problem				90.7
	A bit of a problem				6.2
	A big problem				3.1

A very small proportion of the sample respondents reported problems in their local area in terms of racism, though more reported issues with drunkenness and break-ins. Vandalism, problems with rubbish and graffiti were reported by a larger proportion of the sample. Poor public transport was an area-level problem identified for the largest proportion of the sample, where 65.9% reported this was a big problem in the area, while 15.6% reported this was a bit of a problem. Problems with food shops and problems with open spaces were reported by a very small proportion of the sample.

Table 5 presents the estimation results of area-level deprivation and neighbourhood factors of investigation on the three mental health outcomes (full model results presented in S1-S3 Tables in S1 File). Taking first poor mental health score as measured by MHI-5, for the basic specification (Model 1), living in the most deprived quintile was estimated to increase negative mental health by a score of 3.9 relative to the base category of the least deprived quintile, statistically significant at the 1% level. However, when the model is fully adjusted, in Model 2, the size and significance of this association with poor mental health was attenuated. Residing in an area of middle deprivation/affluence, deprivation quintile three, was estimated to be negatively associated poor mental health score, an association which remained significant across the three model specifications.

**Table 5:** Estimation results on three mental health outcomes.

	Ordinary Least Squares: MHI-5 and EVI4 scores						Logistic regression: Depression		
	Poor mental health: MHI-5			Positive mental health: EVI4			Depression (Marginal effects)		
	Basic model	Full model	Full model with area-level problems principal components	Basic model	Full model	Full model with area-level problems principal components	Basic model	Full model	Full model with area-level problems principal components
Model	(1)	(2)	(3)	(1)	(2)	(3)	(1)	(2)	(3)
**Reference category: Compared to least deprived**									
**Deprivation quintile 1 (Most deprived)**	3.867*** (0.590)	0.727 (0.583)	0.107 (0.579)	-2.964*** (0.746)	0.343 (0.724)	0.960 (0.724)	0.041*** (0.010)	0.000 (0.009)	-0.004 (0.009)
**Deprivation quintile 2**	0.385 (0.541)	-0.615 (0.556)	-0.771 (0.552)	0.279 (0.708)	0.917 (0.698)	1.155* (0.694)	0.017* (0.009)	0.006 (0.010)	0.005 (0.010)
**Deprivation quintile 3**	1.442** (0.560)	0.698 (0.557)	0.503 (0.553)	-0.991 (0.734)	-0.443 (0.706)	-0.134 (0.701)	0.005 (0.009)	-0.002 (0.010)	-0.004 (0.010)
**Deprivation quintile 4**	-0.700 (0.530)	-0.618 (0.516)	-0.676 (0.513)	1.030 (0.715)	0.626 (0.668)	0.744 (0.664)	-0.006 (0.008)	-0.008 (0.010)	-0.009 (0.010)
**Involvement in social clubs and groups**		-2.375*** (0.343)	-2.463*** (0.341)		3.958*** (0.419)	4.068*** (0.417)		-0.011* (0.006)	-0.011* (0.006)
**Principal Component: Area-level safety**			-0.939*** (0.209)			0.726*** (0.244)			-0.005* (0.003)
**Principal Component: Area-level service provision**			-0.183 (0.158)			0.697*** (0.189)			-0.001 (0.002)
**Principal Component: Area-level cleanliness**			-0.428** (0.178)			0.463** (0.200)			-0.002 (0.002)
**N**	7,403	7,403	7,403	7,403	7,403	7,403	7,403	7,403	7,403
**R**^**2**^ **/Log likelihood**	0.02	0.14	0.10	0.03	0.21	0.22	-1745.83	-1480.87	-1474.53

*p<0.1,

**p<0.05,

***p<0.01 denote statistical significance.

Robust standard errors in parentheses. Full results of these models are available in S1-S3 Tables in S1 File.

Model (1) is the most basic specification which additionally controls for gender and age.

Model (2) adjusts further adjusts model (1) to include education level, marital status, employment status, urbanity, whether they have had a long-term illness, self-reported health, medical card status, whether they were an immigrant and whether they were a member of social clubs in their locality.

Model (3) adjusts model (2) to include other area-level variables as a principal component analysis, listed in Table 3. Robustness analyses are presented in S4 Table in S1 File.

Residing in the most deprived quintile of area-level deprivation was associated with a reduction in positive mental health score in the most basic specification, but this association did not remain statistically significant when socioeconomic, health and other individual level factors were accounted for in the adjusted model (Models 2).

In terms of experiences of depression, the estimation results on the most basic model specification estimate that living in the most deprived areas was associated with poorer mental health. However, when the individual’s social, material, and health circumstances were accounted for in Model 2, the estimated effect was nullified.

When other area-level factors were included in the analysis in the form of the first principal components, reported as Model 3 for each of the outcome variables, these are shown to have had a considerable effect on an individual’s mental health. The principal component of ‘area-level safety’ had a significant effect on both positive and negative mental health, while ‘area-level service provision’ was found to have a large significant effect on positive mental health. ‘Area-level cleanliness’ was not estimated to have an impact on the outcome measures. Social connectedness, as indicated by an individual’s participation in clubs or societies was estimated to have a significant positive impact on an individual’s mental health in the adjusted model specifications of Model 2 and Model 3.

Analysis using a binary dependent variable for the mental health scores of a threshold for MHI-5 and EVI also found no discernible effect of area-level deprivation on mental health states, nor that for a Probit analysis approach on the depression outcome as reported in S4 Table in S1 File. In terms of the investigation of differential interaction effects of area-level deprivation with gender and urban areas, few differential associations with mental health indicators were uncovered (results available on request from authors).

## Discussion

This empirical analysis of effect of neighbourhood variables on mental health indicators conducted for the study setting of Ireland, does not find an effect of area-level deprivation on three measures of mental health, when accounting for the socio-economic and health circumstances of an individual. In finding a similar result, Reijneveld and Schene [33] explain that concentrations of problems with mental health in deprived areas are explicable by other social and demographic factors that compose those areas like individual income, and poorer somatic health status.

The findings of this research broadly align with a subset of the literature concerned with the impact of area-level deprivation on mental health. Numerous studies utilising specific measures of mental health have found that area-level deprivation has little or no direct impact on an individual’s mental health when other factors are taken into account (see [21, 35]). This paper therefore lends further weight to a substantial body of research which finds a lack of evidence that area-level deprivation affects the mental health of residents. However, we do find perceptions of aspects of the neighbourhood such as safety, cleanliness, and service provision can influence mental health, which accord with the findings of other studies analysing these particular factors [15, 41, 54]. We find that safety, service provision, and cleanliness are strongly positively associated with the measure of positive mental health, chiming with Leslie and Cairn’s [54] conclusions that neighbourhood aesthetics, crime, and safety may be particularly important perceived environmental factors impacting on residents’ mental health as these factors influence neighbourhood satisfaction. Our results which indicate that poor mental health and depression are inversely associated with better ratings of area-level safety are also in line with evidence from a recent systematic review of the effects of neighbourhood crime on mental health, which identifies potential public health benefits from area-based crime interventions [55]. Furthermore, the importance of involvement in social activities such as membership of clubs also provides credence to similar literature which suggests a positive link between social capital and mental health [54, 56]. More recently, evidence from the US has found that community social capital was associated with lower psychological distress during the COVID-19 pandemic, where it also buffered the harm of the pandemic-induced mobility restrictions [57].

The variation in results reported in the international literature by study setting suggest that the degree to which neighbourhood factors affect mental health appear to be context specific; and in this field of study, one cannot assume a particular direction of effect (or indeed a null effect) for any particular jurisdiction of interest. As a result, empirical investigation of available high-quality data for specific geographies in relation to these issues is required as opposed to assuming the generalisability and applicability of the findings of extant studies in this sphere.

### Strengths and limitations

This research benefits from a large, nationally representative dataset, and the information contained in the survey permits investigation of multiple angles of mental health than is typically examined in other studies in this area. Moreover, rich information on a variety of socio-economic and health related variables contained in the survey allow for controlling for potentially confounding factors in their association with mental health.

A number of limitations of this work must also be acknowledged. The results are based on cross-sectional data which limits the scope for causal inference. The mental health outcomes, as well as the variables concerning area-level problems, are based on self-reported information captured during face-to-face interviews which may be subject to response biases. However, we note that sources of response bias on mental health indicators have not been found to invalidate patterns of observed relationships with demographic variables.

### Policy implications

The findings of this analysis suggest that living in an area characterised as deprived does not appear to present additional risks to mental health beyond social, economic, and health circumstances of the individual. However, the results from the principal component analyses and regression modelling reveal that perceptions of safety and cleanliness in neighbourhoods may be determinants of a person’s mental health. Thus, policies and initiatives which improve safety and cleanliness in neighbourhoods may represent an investment in mental health. Access to services and amenities are associated with reduced poor mental health and greater positive mental state, and so interventions to improve services such as public transport and providing high quality, open spaces may also have advantages for mental health in communities. Social club membership is also found to have a beneficial influence on mental state, suggesting that investment to support engagement with social groups and activities in communities could promote wellbeing.

### Directions for future study

Future studies of the effects of neighbourhoods and mental health may consider aspects such as mental resilience of residents living in deprived areas. Further research is required to understand the precise mechanisms of associations between neighbourhood safety, cleanliness, and service provision and mental health, and whether interventions or improvements to these yield positive impacts. We also note that research in the area of examining the effects of income inequalities on various health outcomes emphasises the need to consider whether associations differ across differ specifications of geographic scales e.g., county-level or State-level, to which this study could not investigate in detail. As such, future studies which benefit from larger sample sizes drawn from various geographical levels and units could better examine whether the effects of deprivation on mental health outcomes differs across alternative spatial scales. Moreover, in light of the changes that have resulted from the COVID-19 pandemic, for example, a greater degree of remote working [58], more evidence is required to understand whether differential effects on mental health have emerged where people are spending greater amounts of time in their residential neighbourhoods for living, working, and leisure. We note that recent research in this field has also called for a greater understanding of the effects of neighbourhood on health over the life course [59]. Existing evidence suggests the effects of exposure to neighbourhood disadvantage can be cumulative, resulting in poorer health later in life, calling for policies which improve neighbourhoods as early in life as possible. Research in this area requires data linkage to longitudinal data.

To conclude, the findings of this investigation suggest that residing in an area characterised by area-level deprivation does not have a statistically significant association with the mental health of adult residents of Ireland across three different measures of mental state. However, social connectedness, as measured by membership of social groups or clubs, is associated with beneficial mental health outcomes. The results also reveal that area safety, cleanliness, and service provision can influence mental health. Such findings may advise that more broad ranging policies and investments in communities, for example in social groups, crime prevention, safety measures and the provision of open spaces and public transport could be advantageous for mental health.

## Supporting information

S1 File Area-level deprivation, neighbourhood factors and associations with mental health. (DOCX)
