# Supplementary material for: Area-level deprivation, neighbourhood factors and associations with mental health
Source: PLoS One. 2023 Jan 30;18(1):e0281146. doi: 10.1371/journal.pone.0281146 (PMC9886251; doi:10.1371/journal.pone.0281146)
Supplement: S1 File — (DOCX) [file pone.0281146.s001.docx]

# Supporting Information File

**Area-level deprivation, neighbourhood factors and associations with mental health**

**Further details on the Haas Pratschke Index**

The deprivation index used in this paper is the Pobal Haas-Pratschke (HP) index of multiple deprivation (Haase and Pratschke 2017). It uses small area datasets to create a multiple deprivation index for the Republic of Ireland. It calculates deprivation scores based on variables which determine demographic profile, social class composition and the labour market situation of the relevant small area. Using Confirmatory Factor Analysis (CFA), the index includes a multitude of variables, the values of which are combined to establish a deprivation valuation for each of the 18,488 small areas in the country (Haase and Pratschke 2017). The variables in each of the domains are outlined as follows:

1. Demographic growth: The Small Area Population (SAP) statistics used to indicate demographic growth are the age dependency rate, population change, proportion of individuals with no or primary education only, proportion of individuals with higher education, number of people per room. This analysis of demographic growth takes account of rural area-level deprivation. Areas of rural decline in Ireland are generally characterised by low population density, reducing population and low levels of education. Therefore, this index takes account of the significant rural population in Ireland and the deprivation therein.
2. Social Class composition: The second group of SAP statistics used by the HP deprivation index provides an indication of social class composition. This includes the proportion of individuals with no or primary education only, the proportion of individuals with tertiary education, the number of individuals per room as well as the proportion of individuals in each social class. This importance of social class is of equal importance to both rural and urban areas. Areas with a traditionally lower social class demographic profile generally have poorer educational attainment, housing and higher unemployment rates.
3. Labour market conditions: The last method by which the HP index takes account of deprivation are the labour market conditions in that area. The labour market situation is an important indicator of urban deprivation. Urban areas with significant unemployment rates generally have significant problems with lower incomes from lack of paid employment. The variables included in this measure are the proportion of lone parents in the area, the proportion of semi and unskilled classes in the area, the male unemployment rate and the female unemployment rate.

**References**

Haase, T., and Pratschke, J. 2017. ‘The 2016 Pobal HP Deprivation Index for Small Areas (SA): Introduction and Reference Tables’.

______________________________________________________

Presentation of full model results (from Table 5 of main manuscript)

**Table S1. Full estimation results: OLS regression on poor mental health (MHI-5).**

| **Outcome: Poor mental health (MHI-5)** | | ***Reference***  ***category*** | **Basic model** | | | | **Full model** | **Full model with area-level problems principal components** |  |
| --- | --- | --- | --- | --- | --- | --- | --- | --- | --- |
| Model | |  | (1) | | | | (2) | (3) |  |
| Deprivation quintile 1  (Most deprived) | | *Least deprived quintile (5)* | 3.867***  (0.590) | | | | 0.727  (0.583) | 0.107  (0.579) |  |
| Deprivation quintile 2 | | *Least deprived quintile* | 0.385  (0.541) | | | | -0.615  (0.556) | -0.771  (0.552) |  |
| Deprivation quintile 3 | | *Least deprived quintile* | 1.442**  (0.560) | | | | 0.697  (0.557) | 0.503  (0.553) |  |
| Deprivation quintile 4 | | *Least deprived quintile* | -0.700  (0.530) | | | | -0.618  (0.516) | -0.676  (0.513) |  |
| Male | | *Female* | -2.548***  (0.360) | | | | -2.186***  (0.356) | -2.172***  (0.354) |  |
| Age 25-44 | | *Age 18-24* | -1.992***  (0.741) | | | | -0.092  (0.819) | 0.431  (0.810) |  |
| Age 45-64 | | *Age 18-24* | -2.089***  (0.741) | | | | -1.187  (0.809) | -0.553  (0.800) |  |
| Age 65+ | | *Age 18-24* | -2.318***  (0.760) | | | | -3.164***  (0.925) | -2.198  (0.919) |  |
| \| Secondary educated \| \| --- \| | | *Primary educated* |  | | | | -1.002  (0.678) | -0.908  (0.673) |  |
| \| Tertiary educated \| \| --- \| | | *Primary educated* |  | | | | -0.910  (0.742) | -0.961  (0.735) |  |
| \| Immigrant \| \| --- \| \|  \| | | *Not an immigrant* |  | | | | 0.499  (0.504) | 0.514  (0.497) |  |
| \| Married \| \| --- \| | | *Not married* |  | | | | -2.525***  (0.375) | -2.560***  (0.373) |  |
| \| Private health insurance \| \| --- \| | | *No private health insurance* |  | | | | -1.400***  (0.407) | -1.282***  (0.405) |  |
| GP visit card holder | | *No medical/GP card* |  | | | | -1.262*  (0.733) | -1.349*  (0.731) |  |
| Medical card holder | | *No medical/GP card* |  | | | | 0.365  (0.486) | 0.286  (0.483) |  |
| Urban | | *Rural* |  | | | | 1.350***  (0.367) | 0.995**  (0.388) |  |
| \| Good or better self-rated health \| \| --- \| \|  \| | | *Fair, poor or very poor self-rated health* |  | | | | -8.740***  (0.614) | -8.822***  (0.611) |  |
| Employed | | *Unemployed* |  | | | | 3.484  (0.896) | 3.176***  (0.885) |  |
| Home maker | | *Unemployed* |  | | | | -0.652  (0.613) | -0.653  (0.613) |  |
| Retired | | *Unemployed* |  | | | | 2.299***  (0.517) | 2.379***  (0.515) |  |
| Long term illness | | *No long-term illness* |  | | | | 3.765***  (0.446) | 3.507***  (0.446) |  |
| Involvement in social clubs and groups | | *No involvement in social clubs and groups* |  | | | | -2.375***  (0.343) | -2.463***  (0.341) |  |
| \| Area-level safety \| \| --- \| | |  |  | |  | | | -0.939***  (0.209) |  |
| Area-level service provision | |  |  | |  | | | -0.183  (0.158) |  |
| \| Area-level cleanliness \| \| --- \| \|  \| | |  |  | |  | | | -0.428**  (0.178) |  |
| Intercept |  | | | 20.3***  (0.776) | | 28.7***  (1.297) | | 28.7***  (1.287) |  |
| N |  | | | 7,403 | | 7,403 | | 7,403 |  |
| R^2^ |  | | | 0.01 | | 0.14 | | 0.16 |  |
| *p<0.1, **p<0.05, ***p<0.01 denote statistical significance. Robust standard errors in parentheses. | | | | | | | | | |

**Table S2. Full estimation results: OLS regression on positive mental health (EVI).**

| **Outcome: Positive mental health (EVI)** | | ***Reference category*** | **Basic model** | | | | **Full model** | **Full model with area-level problems principal components** |  |
| --- | --- | --- | --- | --- | --- | --- | --- | --- | --- |
| Model | |  | (1) | | | | (2) | (3) |  |
| Deprivation quintile 1  (Most deprived) | | *Least deprived quintile (5)* | -2.964***  (0.746) | | | | 0.343  (0.724) | 0.960  (0.724) |  |
| Deprivation quintile 2 | | *Least deprived quintile* | 0.279  (0.708) | | | | 0.917  (0.698) | 1.155*  (0.694) |  |
| Deprivation quintile 3 | | *Least deprived quintile* | -0.991  (0.734) | | | | -0.443  (0.706) | -0.134  (0.701) |  |
| Deprivation quintile 4 | | *Least deprived quintile* | 1.030  (0.715) | | | | 0.626  (0.668) | 0.744  (0.664) |  |
| Male | | *Female* | 4.545***  (0.453) | | | | 4.236***  (0.427) | 4.200***  (0.425) |  |
| Age 25-44 | | *Age 18-24* | -0.416  (0.869) | | | | -0.158  (0.925) | -0.585  (0.917) |  |
| Age 45-64 | | *Age 18-24* | -0.693  (0.870) | | | | 2.043**  (0.917) | 1.286  (0.911) |  |
| Age 65+ | | *Age 18-24* | -2.615***  (0.902) | | | | 3.041***  (1.070) | 1.921*  (1.074) |  |
| \| Secondary educated \| \| --- \| | | *Primary educated* |  | | | | 1.531**  (0.761) | 1.457*  (0.764) |  |
| \| Tertiary educated \| \| --- \| | | *Primary educated* |  | | | | 0.879  (0.855) | 0.978  (0.855) |  |
| \| Immigrant \| \| --- \| \|  \| | | *Not an immigrant* |  | | | | 1.684***  (0.585) | 1.687***  (0.580) |  |
| \| Married \| \| --- \| | | *Not married* |  | | | | 0.397  (0.451) | 0.496  (0.449) |  |
| \| Private health insurance \| \| --- \| | | *No private health insurance* |  | | | | 0.902*  (0.514) | 0.773  (0.510) |  |
| GP visit card holder | | *No medical/GP card* |  | | | | 1.004  (0.894) | 1.111  (0.890) |  |
| Medical card holder | | *No medical/GP card* |  | | | | -0.190  (0.588) | -0.070  (0.583) |  |
| Urban | | *Rural* |  | | | | -2.378***  (0.444) | -2.432***  (0.466) |  |
| \| Good or better self-rated  health \| \| --- \| \|  \| | | *Fair, poor or very poor self-rated health* |  | | | | 15.409***  (0.703) | 15.487***  (0.704) |  |
| Employed | | *Unemployed* |  | | | | 0.831  (0.962) | 1.203  (0.957) |  |
| Home maker | | *Unemployed* |  | | | | 1.598**  (0.737) | 1.577**  (0.737) |  |
| Retired | | *Unemployed* |  | | | | -1.250**  (0.603) | -1.383**  (0.601) |  |
| Long term illness | | *No long-term illness* |  | | | | -7.546***  (0.548) | -7.186***  (0.550) |  |
| Involvement in social clubs and groups | | *No involvement in social clubs and groups* |  | | | | 3.958***  (0.419) | 4.068***  (0.417) |  |
| \| Area-level safety \| \| --- \| | |  |  | |  | | | 0.726***  (0.244) |  |
| Area-level service provision | |  |  | |  | | | 0.697***  (0.189) |  |
| \| Area-level cleanliness \| \| --- \| \|  \| | |  |  | |  | | | 0.463**  (0.200) |  |
| Intercept |  | | | 65.7***  (0.947) | | 50.0***  (1.525) | | 50.2  (1.516) |  |
| N |  | | | 7,403 | | 7,403 | | 7,403 |  |
| R^2^ |  | | | 0.02 | | 0.21 | | 0.22 |  |
| *p<0.1, **p<0.05, ***p<0.01 denote statistical significance. Robust standard errors in parentheses. | | | | | | | | | |

**Table S3. Full estimation results: Logistic regression on experience with depression in previous 12 months.**

| **Outcome: reported suffering from depression in previous 12 months** | | ***Reference***  ***category*** | **Basic model** | | | | **Full model** | **Full model with area-level problems principal components** |  |
| --- | --- | --- | --- | --- | --- | --- | --- | --- | --- |
| Model | |  | (1) | | | | (2) | (3) |  |
| Deprivation quintile 1  (Most deprived) | | *Least deprived quintile (5)* | 0.041***  (0.010) | | | | 0.000  (0.009) | -0.004  (0.009) |  |
| Deprivation quintile 2 | | *Least deprived quintile* | 0.017*  (0.009) | | | | 0.006  (0.010) | 0.005  (0.010) |  |
| Deprivation quintile 3 | | *Least deprived quintile* | 0.005  (0.009) | | | | -0.002  (0.010) | -0.004  (0.010) |  |
| Deprivation quintile 4 | | *Least deprived quintile* | -0.006  (0.008) | | | | -0.008  (0.010) | -0.009  (0.010) |  |
| Male | | *Female* | -0.016  (0.006) | | | | -0.013**  (0.006) | -0.013**  (0.006) |  |
| Age 25-44 | | *Age 18-24* | 0.005  (0.011) | | | | 0.021*  (0.012) | 0.024**  (0.012) |  |
| Age 45-64 | | *Age 18-24* | 0.022  (0.011) | | | | 0.012  (0.012) | 0.016  (0.011) |  |
| Age 65+ | | *Age 18-24* | 0.001  (0.011) | | | | -0.018  (0.013) | -0.012  (0.012) |  |
| \| Secondary educated \| \| --- \| | | *Primary educated* |  | | | | -0.005  (0.009) | -0.004  (0.009) |  |
| \| Tertiary educated \| \| --- \| | | *Primary educated* |  | | | | -0.002  (0.011) | -0.002  (0.011) |  |
| \| Immigrant \| \| --- \| \|  \| | | *Not an immigrant* |  | | | | 0.000  (0.008) | 0.000  (0.008) |  |
| \| Married \| \| --- \| | | *Not married* |  | | | | -0.027***  (0.006) | -0.027***  (0.006) |  |
| \| Private health insurance \| \| --- \| | | *No private health insurance* |  | | | | -0.006  (0.008) | -0.005  (0.008) |  |
| GP visit card holder | | *No medical/GP card* |  | | | | -0.025**  (0.011) | -0.026**  (0.011) |  |
| Medical card holder | | *No medical/GP card* |  | | | | 0.015**  (0.008) | 0.014*  (0.008) |  |
| Urban | | *Rural* |  | | | | 0.021***  (0.006) | 0.019***  (0.007) |  |
| \| Good or better self-rated  health \| \| --- \| \|  \| | | *Fair, poor or very poor self-rated health* |  | | | | -0.047***  (0.007) | -0.048***  (0.007) |  |
| Employed | | *Unemployed* |  | | | | 0.042***  (0.014) | 0.040***  (0.014) |  |
| Home maker | | *Unemployed* |  | | | | -0.009  (0.008) | -0.010  (0.008) |  |
| Retired | | *Unemployed* |  | | | | 0.032***  (0.008) | 0.032***  (0.008) |  |
| Long term illness | | *No long-term illness* |  | | | | 0.072***  (0.007) | 0.070***  (0.007) |  |
| Involvement in social clubs and groups | | *No involvement in social clubs and groups* |  | | | | -0.011*  (0.006) | -0.011*  (0.006) |  |
| \| Area-level safety \| \| --- \| | |  |  | |  | | | -0.005*  (0.003) |  |
| Area-level  service provision | |  |  | |  | | | -0.001  (0.002) |  |
| \| Area-level cleanliness \| \| --- \| \|  \| | |  |  | |  | | | -0.002  (0.002) |  |
| N |  | | | 7,403 | | 7,403 | | 7,403 |  |
| Log likelihood |  | | | -1745.8 | | -1480.9 | | -1474.5 |  |
| *p<0.1, **p<0.05, ***p<0.01 denote statistical significance. Robust standard errors in parentheses. | | | | | | | | | |

**Table S4. Estimation results for alternative binary outcome models.**

|  | **Logistic regression: MHI-5 and EVI4 scores threshold of 80** | | | | | | **Probit regression: Depression** | | |
| --- | --- | --- | --- | --- | --- | --- | --- | --- | --- |
|  | **Poor mental health: MHI-5 threshold (Marginal effects)**  **(Scale reversed to indicate poor mental health)** | | | **Positive mental health: EVI4 threshold**  **(Marginal effects)** | | | **Depression (Marginal effects)** | | |
|  | **Basic model** | **Full model** | **Full model with area-level problems principal components** | **Basic model** | **Full model** | **Full model with area-level problems principal components** | **Basic model** | **Full model** | **Full model with area-level problems principal components** |
| **Model** | **(1)** | **(2)** | **(3)** | **(1)** | **(2)** | **(3)** | **(1)** | **(2)** | **(3)** |
| **Reference category: *Least deprived quintile (5)*** | | | | | | | | | |
|  |  |  |  |  |  |  |  |  |  |
| **Deprivation quintile 1**  **(Most deprived)** | 0.088***  (0.019) | 0.011  (0.019) | -0.006  (0.019) | -0.014  (0.015) | 0.019  (0.016) | 0.027*  (0.016) | 0.042***  (0.010) | 0.005  (0.009) | 0.001  (0.009) |
| **Deprivation quintile 2** | \| -0.005 \| \| --- \|   (0.018) | -0.035*  (0.019) | -0.040**  (0.019) | 0.007  (0.015) | 0.015  (0.015) | 0.019  (0.015) | 0.017*  (0.009) | 0.009  (0.009) | 0.008  (0.009) |
| **Deprivation quintile 3** | 0.026  (0.019) | 0.003  (0.019) | -0.003  (0.019) | 0.005  (0.015) | 0.012  (0.015) | 0.017  (0.015) | 0.006  (0.009) | 0.002  (0.009) | 0.000  (0.009) |
| **Deprivation quintile 4** | -0.031*  (0.018) | -0.033*  (0.018) | -0.036*  (0.018) | 0.028*  (0.016) | 0.025*  (0.015) | 0.026*  (0.015) | -0.006  (0.008) | -0.002  (0.009) | -0.003  (0.009) |
| **Involvement in social clubs and groups** |  | -0.267*** (0.053) | -0.057***  (0.011) |  | 0.054***  (0.009) | 0.056***  (0.009) |  | -0.010*  (0.006) | -0.010*  (0.006) |
| **Principal Component: Area-level safety** |  |  | -0.060***  (0.011) |  |  | 0.006  (0.005) |  |  | -0.004*  (0.003) |
| **Principal Component: Area-level service provision** |  |  | -0.008*  (0.005) |  |  | 0.014***  (0.004) |  |  | -0.002  (0.002) |
| **Principal Component: Area-level cleanliness** |  |  | 0.012**  (0.005) |  |  | 0.009**  (0.004) |  |  | -0.002  (0.002) |
| **N** | 7,403 | 7,403 | 7,403 | 7,403 | 7,403 | 7,403 | 7,403 | 7,403 | 7,403 |
| **Log likelihood** | -4887.26 | -4590.38 | -4555.62 | -3710.57 | -3493.03 | -3476.343 | -1745.67 | -1479.95 | -1472.79 |
| *p<0.1, **p<0.05, ***p<0.01 denote statistical significance. Robust standard errors in parentheses. Robust standard errors in parentheses.  Model (1) is the most basic specification which additionally controls for gender and age.  Model (2) adjusts further adjusts model (1) to include education level, marital status, employment status, urbanity, whether they have had a long-term illness, self-reported health, medical card status, whether they were an immigrant and whether they were a member of social clubs in their locality.  Model (3) adjusts model (2) to include other area-level variables as a principal component analysis, listed in Table 3 of main paper. | | | | | | | | | |
